# Supplementary material for: Estimation of serum C-reactive protein activity in periodontal health and disease and response to treatment: a clinico-biochemical study
Source: PeerJ. 2023 Dec 4;11:e16495. doi: 10.7717/peerj.16495 (PMC10702329; doi:10.7717/peerj.16495)
Supplement: Supplemental Information 2 [file peerj-11-16495-s002.docx]

**Case History Proforma**

**Case no: Date:**

Age:

Sex:

Address:

Chief complaint:

Medical history:

Past Dental history pertaining to periodontal treatment:

Teeth cleaning habits:

Any other habits:

Oral Hygiene status: Poor/ fair/ Good

**Examination of the gingiva:**

- Colour
- Contour
- Consistency
- Size
- Surface texture
- Form & Position
- Bleeding on probing
- Suppuration

Pocket depth (mm)

|  |  |  |  |  |  |  |  |  |  |  |  |  |  |
| --- | --- | --- | --- | --- | --- | --- | --- | --- | --- | --- | --- | --- | --- |
| **17** | **16** | **15** | **14** | **13** | **12** | **11** | **21** | **22** | **23** | **24** | **25** | **26** | **27** |
| **47** | **46** | **45** | **44** | **43** | **42** | **41** | **31** | **32** | **33** | **34** | **35** | **36** | **37** |
|  |  |  |  |  |  |  |  |  |  |  |  |  |  |

Plaque Index

Gingival Index

|  |  |  |  |  |  |  |  |  |  |  |  |  |  |
| --- | --- | --- | --- | --- | --- | --- | --- | --- | --- | --- | --- | --- | --- |
| **17** | **16** | **15** | **14** | **13** | **12** | **11** | **21** | **22** | **23** | **24** | **25** | **26** | **27** |
| **47** | **46** | **45** | **44** | **43** | **42** | **41** | **31** | **32** | **33** | **34** | **35** | **36** | **37** |
|  |  |  |  |  |  |  |  |  |  |  |  |  |  |

|  |  |  |  |  |  |  |  |  |  |  |  |  |  |
| --- | --- | --- | --- | --- | --- | --- | --- | --- | --- | --- | --- | --- | --- |
| **17** | **16** | **15** | **14** | **13** | **12** | **11** | **21** | **22** | **23** | **24** | **25** | **26** | **27** |
| **47** | **46** | **45** | **44** | **43** | **42** | **41** | **31** | **32** | **33** | **34** | **35** | **36** | **37** |
|  |  |  |  |  |  |  |  |  |  |  |  |  |  |

Russel’s Periodontal Index

|  |  |  |  |  |  |  |  |  |  |  |  |  |  |
| --- | --- | --- | --- | --- | --- | --- | --- | --- | --- | --- | --- | --- | --- |
| **17** | **16** | **15** | **14** | **13** | **12** | **11** | **21** | **22** | **23** | **24** | **25** | **26** | **27** |
| **47** | **46** | **45** | **44** | **43** | **42** | **41** | **31** | **32** | **33** | **34** | **35** | **36** | **37** |
|  |  |  |  |  |  |  |  |  |  |  |  |  |  |

**Investigations**

Serum C-Reactive Protein Level

**Clinical Diagnosis**
